# Prediction of Communication Effectiveness During Media Skills Training Using Commercial Automatic Non-verbal Recognition Systems

**DOI:** 10.3389/fpsyg.2021.675721

**Published:** 2021-09-29

**Authors:** Monica Pereira, Hongying Meng, Kate Hone

**Affiliations:** ^1^Department of Psychology, School of Social Sciences, London Metropolitan University, London, United Kingdom; ^2^Department of Electronic and Computer Engineering, College of Engineering, Design and Physical Sciences, Brunel University London, London, United Kingdom; ^3^Department of Computer Science, College of Engineering, Design and Physical Sciences, Brunel University London, London, United Kingdom

**Keywords:** social signals detection, commercial technologies, communication skills, training, non-verbal signals, media interviews, multimodal fusion

## Abstract

It is well recognised that social signals play an important role in communication effectiveness. Observation of videos to understand non-verbal behaviour is time-consuming and limits the potential to incorporate detailed and accurate feedback of this behaviour in practical applications such as communication skills training or performance evaluation. The aim of the current research is twofold: (1) to investigate whether off-the-shelf emotion recognition technology can detect social signals in media interviews and (2) to identify which combinations of social signals are most promising for evaluating trainees’ performance in a media interview. To investigate this, non-verbal signals were automatically recognised from practice on-camera media interviews conducted within a media training setting with a sample size of 34. Automated non-verbal signal detection consists of multimodal features including facial expression, hand gestures, vocal behaviour and ‘honest’ signals. The on-camera interviews were categorised into effective and poor communication exemplars based on communication skills ratings provided by trainers and neutral observers which served as a ground truth. A correlation-based feature selection method was used to select signals associated with performance. To assess the accuracy of the selected features, a number of machine learning classification techniques were used. Naive Bayes analysis produced the best results with an F-measure of 0.76 and prediction accuracy of 78%. Results revealed that a combination of body movements, hand movements and facial expression are relevant for establishing communication effectiveness in the context of media interviews. The results of the current study have implications for the automatic evaluation of media interviews with a number of potential application areas including enhancing communication training including current media skills training.

## Introduction

Skilful communication in media interviews is important in a range of organisations and job roles. Significant training investments are made to improve communication skills so that that relevant employees come across positively to the media. Communication is a complex phenomenon that is defined as the transmission of information from one person to another (Fiske, 2010; Knapp et al., 2013; Deveugele, 2015). Early research in psychology has suggested that verbal communication only accounts for 7% of social perception (Vinciarelli et al., 2009). However, the weight of messages depends on the context and the type of social interaction. It is therefore important that accurate and objective observations of non-verbal cues are incorporated into assessment of media performance and training interventions to improve performance. However, current tools to support this are limited.

Earlier research in the field of non-verbal analysis relied solely on meticulous observation and analysis of video data, such as viewing hours of recorded videos in order to interpret social situations (Vrij et al., 2000; Hart et al., 2016). This method of behaviour analysis is prone to subjectivity, is time consuming and does not scale with large amounts of data. In this paper, we propose a scalable alternative that gives rise to the possibility of faster, easily accessible to researchers for evaluating emotions and more objective measurement of non-verbal signals for professionals. Specifically, we explore the potential of a range of off-the-shelf-commercial-technologies, recognising a range of non-verbal signals, to identify skilful performance in the context of media interviews. Off-the-shelf-commercial-technologies have been proposed to be an effective means of detecting non-verbal signals in the wild (Dupré et al., 2018; Pereira and Hone, 2021).

Researchers chose to use off-the-shelf-commercial-technologies rather than develop bespoke solutions in order to provide relatively rapid proof of concept for the relevance of a range of channels in the evaluation of media skills performance. This approach was also taken to allow rapid transferability to end users, since the potential technologies can already be obtained commercially. The rationale is to help narrow the design space for future bespoke solutions. In addition, the focus is on functional applicability of solutions developed using affect technology. This could be beneficial as this technology enables the user/researcher to process recordings/images locally where classification of emotions and expressions are extracted and produced by the software’s classifier (Dupré et al., 2018).

In the current study, participants that took part in interviews during media skills training which were recorded. Data was collected using several technologies to allow the detection of emotion and non-verbal behaviours. The interviews were also assessed for communication skills quality by trainers and neutral observers using a standardised survey instrument. Analysis focussed on exploring which of the detected signals were associated with good vs. poor performance as rated by human observers and conclusions are drawn regarding the potential future use of such technologies.

To the researcher’s knowledge there have been no studies investigating whether commercial technology can detect relevant multimodal social signals for effective communication and no studies investigating communication in the context of media interviews. Therefore, the aim of this twofold: (1) to investigate whether commercial automated affect recognition technology can detect non-verbal signals in a dyadic interaction and (2) to investigate which combination of multimodal signals are necessary for effective communication in a media interview.

Therefore, the research question (RQ) is to be investigated:

Which combination of non-verbal signals are necessary for evaluating communication skill performance during a media interview?

The RQ is explored using the data from a range of practice media interviews during media training workshops. The current research provides four main contributions:

(1) It provides a deeper understanding of communication skills,
(2) It provides evidence that the use of this type of automated technology can be used to detect social and non-verbal signals in a person–person context,
(3) Identifies the relevant signals for media interviews,
(4) Assists trainers in choosing the best type of technology to use in training to improve performance outputs.

## Background

In this section we briefly review previous work on non-verbal signals before considering the role of non-verbal signals in the specific case of media interviews which forms the focus of this paper. We then briefly introduce research using automated detection of non-verbal signals and limitations in previous research; finally, we introduce the reader to the aims and objectives of the current research.

### Non-verbal Communication

The complexities of communication lie in the functions of the context and relationship. To understand communication it needs to be acknowledged that communication is multimodal (Hunyadi, 2019). There is a large body of evidence that non-verbal signals are important across many types of human interaction (see Knapp et al., 2013). Studies of non-verbal signals show that communication is typically characterised by the complex interplay of reciprocal signals between interlocutors (Knapp et al., 2013). A number of non-verbal signals are thought to correspond to emotions felt internally which are expressed consciously or unconsciously. In evolutionary terms, displaying emotions benefits both senders and receivers in social interactions. These signals are communicated via multiple channels; such as facial expressions, vocal behaviour (i.e., tone of voice and vocal bursts), gestures and posture (Adams and Kveraga, 2015).

The human face contains a multitude of different functions. One of these functions is to express emotions. From the early work of Darwin (2015) to later empirical work by Ekman et al. (1969) and Ekman and Friesen (1971) there have been numerous suggestions for the existence of universal (recognised across all cultures) basic emotions that are displayed in recognisable facial expressions.

Ekman identified six basic emotions; anger, fear, disgust, happiness, sadness, and surprise and seven universally recognised facial expressions encompassing contempt as well as the six basic emotions (Ekman and Friesen, 1986). Other theories have proposed various other basic emotions; examples include anxiety shame and pleasure (Ortony and Turner, 1990). However, this theory has been argued to be reductionist and simplistic (Gross and Feldman-Barrett, 2011). However, research in emotion continues to apply this theory (see Ekman, 2016).

Ekman (1997) developed a manual system for labelling facial actions. This system is called the Facial Action Coding System (FACS). This system is based on the mapping of muscles on the face to different facial expressions and defines a total of 18 Action Units (AUs) in the lower face, 9 in the upper face, 9 for eye position, 11 for head position, and 14 miscellaneous movements. Human coders use this system to manually code all facial expressions. As these AUs are independent of interpretations they can be used in the recognition of basic emotions (EMFACS). For example, the AUs involved in an emotional display of *happiness* are Action Units 6 (Cheek raiser) and 12 (Lip corner puller).

Vocal non-verbal behaviour contains all the cues surrounding verbal messages which influence the meaning of spoken content. There are five major components to vocal non-verbal behaviour which include linguistic vocalisations, non-linguistic vocalisations, voice quality, silence and turn-taking. Each of these contribute to the social perception of a message (Hall et al., 2019). For example, the vocal intonation can change the tone of a message to be ironic or sarcastic.

Voice quality relates to prosodic features such as pitch, energy and tempo. This accounts for *how* something is said. It conveys emotions such as anger or fear. These two emotions are displayed by shouting (Lieberman, 1976). Pitch influences perception of dominance and extraversion, fluency relates to persuasiveness (Vinciarelli et al., 2009). Linguistic vocalisations are non-words that are used in place of words such as *uhm* or *ah ha.* These are called segregates which are often used in social situations when embarrassed or have difficulty with a social interaction (Glass et al., 1982). Non-linguistic vocalisations include outbusts such as crying, groaning, laughing, or sobbing. Crying, for instance, is often coupled with mirroring (Chartrand and Bargh, 1999) which enhances social bonds.

Gestures are often used to regulate interactions by changing arm movement, postures and kinematics to display emotions (Pollick et al., 2001; Gross et al., 2012). For example, thumbs up to indicate acknowledgement (Altman, 1978). Gestures can also be used to display unconscious information such as the use of *adaptors* such as folding arms or rhythmically moving legs to indicate boredom (Pentland and Heibeck, 2010). Postures are also assumed consciously or unconsciously as they tend to reveal the attitudes of people toward a social situation (Scheflen, 1964).

It is known that communication between two interlocutors depends on the goal and the context. For instance, non-verbal signals that have been detected and identified as potentially important in a job interview are to smile more (Naim et al., 2016), whereas in a healthcare setting turn-taking, speaking ratio, volume, pitch, smiling, frowning, head tilting, nodding, shaking and overall body movements were extracted (Liu et al., 2016). In the classroom, non-verbal cues that were extracted during presentations were prosody, voice quality and gesturing activity (Cheng et al., 2014). These studies suggest that appropriate displays of non-verbal signals differ depending on the context.

Overall there is very rich literature on the role of non-verbal signals in effective communication. This section has briefly described some of the main channels of non-verbal communication and highlights the importance of looking at signals within the context of reciprocal exchanges. Communication context is also important, so we now consider the specific communication context of media interviews which forms the basis of this study.

### Non-verbal Communication in Media Interviews

Media training manuals typically suggest some specific behaviours which should be avoided in media interviews. Behaviours such as lack of vocal conviction, lack of eye contact, fast speaking rate, monotone voice and hesitation are an indication of nervousness, uncertainty and boredom and influence how the interviewee is perceived by the audience (Taylor, 2015). An additional behaviour that can be interpreted as boredom is excessive movements such as swaying and rocking, particularly when the other person in speaking (Tao and Tan, 2009).

Combinatorial signals are likely to be important for a good media interview; such as mirroring the interviewer’s movements, maintaining eye-contact and smiling. Together, these signals suggest that the interviewee is listening, signposts turn-taking in conversation (Ho et al., 2015; Taylor, 2015), illustrates confidence, honesty, and dominance (Knutson, 1996; Lapidot-Lefler and Barak, 2012).

There are a limited number of studies which have empirically explored the relationships between observable non-verbal behaviours and observer subjective judgments within the context of media interviews. Such studies typically focus on small samples of interviews with high profile interviewees such as politicians. For example, Babad (1999) correlated observer judgement of global impression (positive/negative) created in a media interview with a set of observer judgements in relation to observable behaviour. This paper contained three studies which focussed on the behaviours of six interviewees taking part in televised political interviews and found several common patterns across these individuals. The behaviours which appeared to create a positive impression included smiling, a relaxed face, nodding and round hand movements. Conversely the behaviours associated with negative judgements included beating hand movements, leaning forward and blinking. Studies such as this have typically been small scale given the challenge of hand coding the non-verbal communicative behaviours under study. However, the development of technologies to automatically detect non-verbal signals presents increased opportunity to develop an understanding of the cues that are associated with creating a positive impression in a media interview.

While non-verbal cues are generally accepted to be an important element within media interviews and are typically included within training, the accuracy of trainers in detecting these signals is uncertain as inference of emotions is subjective by nature (Vrij et al., 2000).

### Automated Detection of Non-verbal Signals

Technology developments within the fields of affective computing and social signals processing (SSP) in recent years have allowed the automatic detection of a range of non-verbal signals. Vinciarelli et al. (2012) provide an in-depth survey of SSP and a review of affect detection systems can be found in D’mello and Kory (2015). SSP is a research area that models human–human interaction to develop emotionally intelligent machines.

From the SSP perspective, Pentland has proposed that this interplay of vocal behaviour, turn-taking, movement and posture in social interactions represents a second channel of communication which he coined the term *honest signals* (Pentland and Heibeck, 2010). These signals, which he identifies as mimicry (mirroring – Chameleon Effect), influence, activity and consistency, are proposed to be evolutionarily important predictors of communication partner characteristics and intentions (Bilakhia et al., 2015). Sung and Pentland (2005) and Curhan and Pentland (2007) provide a number of empirical examples where honest signals predict communication task outcomes.

Several studies have used such technology to investigate non-verbal communication in a range of interactions. These include medical settings (Hart et al., 2016), job interviews (Frauendorfer et al., 2014; Naim et al., 2016), teaching (Chen et al., 2011, 2015; Bahreini et al., 2016), and improving social communication in individuals with autism (Bernardini et al., 2014; Chen et al., 2016). However, we are not aware of previous research with automated technology specifically considering the social signals that are required for effective communication in a media interview.

Typically, research in SSP domain focuses on single channels or signals of a single individual rather than reciprocal signals (Kim and Suzuki, 2014). The reciprocal exchange of signals between a sender and a receiver is important to observe as this interchange influences behaviour. In addition to this, research often relies on general indications from the literature of what signals represent a good performance in the communication task rather than defining ‘good’ in relation to the specific set of signals detected by the technology.

Rasipuram and Jayagopi (2018) predict communication performance by capturing multimodal channels during a face–face interview and an interface interview. Signals captured included movements, facial expression, hand gestures, posture, eye contact, verbal features and attention. Researchers found that participants had an optimal rate of speech and communicated better in face-face interview than the interface interview. This finding suggests that communication is better when multiple signals can be seen in an interaction by both interlocutors. This is consistent with Adams and Kveragas’ theory that visual integration of combinations of social cues is necessary for behaviourally adapting in responding to others (Adams and Kveraga, 2015).

More recently, off-the-shelf-commercial technology has been developed and is made available to all users and enables users to produce data locally using the classifier made available (Dupré et al., 2018). This enables users to easily access their data. Some examples of these technologies are Emotients FACET or Affectiva that capture facial expressions (Stöckli et al., 2018), Microsoft Kinect to capture body movements (Barmaki, 2016), Sociometric Badges to measure interactions between two or more people (Zhang et al., 2018a) and obtaining movement of hands using accelerometers (Koskimäki et al., 2017).

The successful detection of social signals associated with a good performance in a media interview using this technology could have many potential applications. Firstly, it has potential to improve the quality of performance feedback in training to support a human trainer, since trainers may not be able to observe and consider all the cues that may impact effective communication and individual performance is currently highly dependent on the trainer’s experience (Aspegren, 1999). Secondly, it can objectively select the social signals that are required for effective communication in a number of social interactions (Naim et al., 2016).

### The Current Research

In sum, contexts in which social signals have been investigated are job interviews, public speaking and in the classroom (Bahreini et al., 2017). Previous research is limited to unimodal analysis of social interactions, but more recent research provides evidence that a multimodal approach is more effective at synthesizing and interpreting social interactions. There is little to no research investigating the appropriate social signals for effective communication in media skills training which is important due to the nature of communication in this setting.

The aim of this paper are twofold:

(1) Investigate which combinations of signals are relevant in a media interview.
(2) Present a possible more objective method of capturing social signals during media interviews as opposed to traditional methods of watching a video.

We conducted a study to investigate the signals associated with good media skills interview performance by automatically detecting a variety of social signals (including reciprocal behaviour in relation to the interviewer) during the context of media training exercises and looked at how these predicted good and bad performance as judged by human raters.

## Materials and Methods

The current research applied automatic detection of social signals in an on-camera, face-to-face interview. This section details the study design (see section “Study Design”), participant characteristics (see section “Participants”), the technology used to capture social signals during interviews (see section “Off-the-Shelf Non-verbal Signal Detection Technology”), how performance was rated by human raters (see section “Subjective Measures of Communication Skills”) and how the data was collected describing the procedure together with the study layout (see section “Procedure and Media Skills Workshop Details”).

### Study Design

The current research explored a dyadic interaction during a media interview setting where participants were interviewed by a journalist in face-face on-camera media interviews. Signals which were automatically detected were facial expression, vocal signals, ‘honest signals’ and hand gestures. Communication performance during interviews were judged by human raters. Subsequently, using these ratings, interviews were categorised into effective and poor communicators. The data was then explored to identify relationships between signals captured and human judgements of performance. The data was further explored to identify relationships between detected signals and human judgements. Details of these can be seen in the following sections.

### Participants

A total of 39 participants were recruited to take part in media interview training at a London University (17 males and 22 females; age ranged from 18 to 56). All participants were research students or research staff and none had a social impairment.

A total of two workshops were conducted, the first contained 17 participants (11 males and 6 females; age ranged from 18 to 65 years old) which included nine participants who were native English speakers (participants who declared that English was their first language) and 10 participants who were non-native English speakers (participants who declared that English was not their first language). Experience in public speaking ranged from ‘none’ to ‘extensive and experience in media interviews ranged from ‘none’ to ‘some.’ The roles that participants had within the university in the first workshop included research staff (5), research student (10), professional staff (1), and taught student (1).

The second workshop included 22 participants (6 males and 16 females; age ranged from 18 to 55 years old) which included 6 native English speakers and 16 non-native English speakers. Experience in public speaking ranged from no experience to extensive and experience in media interviews ranged from none to some experiences. The roles that participants had within the university in the second workshop included taught students (3), research staff (1), and research students (18).

### Off-the-Shelf Non-verbal Signal Detection Technology

Non-verbal signals that were detected throughout the duration of the interviews included vocal signals, honest signals, facial expressions, and hand movements. This section introduces the commercial technology used to capture these signals. The accuracies will be reported using measures of Receiver Operating Characteristics (ROC). The ROC measure demonstrates the diagnostic ability of a system based on a curve created by the true positive rate against the false positive rate. The closer the ROC score is to 1 the more accurate the classifier suggesting that the technology measures what is suggests that it measures (Macmillan and Creelman, 2004).

#### Vocal Behaviour Detection

Nemesysco Ltd’s QA5 technology was used to detect vocal signals of participants during interviews. This software uses proprietary signal processing algorithms to extract parameters from the voice and classify according to a range of vocal signals^1^. Table 1 summarises the emotions that the technology claims to classify with a brief description of each one.

**TABLE 1:** Definitions of emotion labels produced by Nemesysco/Layered voice analysis.

Emotion	Description
Energy	Indicates if speaker is sad, tired, boredom, comfortable or highly energetic.
Content	Indicates how pleased or happy a person is
Upset	Indicates how unpleased or sad a person is
Angry	Indicates how angry a person is
Stressed	Indicates how nervous a person is
Embarrassment	Indicates how uncomfortable a person is
Intensive thinking	Indicates thinking intensity while speaking
Imagination Activity	Indicates whether the person is recalling information or visualising something
Hesitation	Indicates how comfortable a person is when making the statement
Uncertainty	Indicates how certain or uncertain a person is
Excitement	Indicates how positively or negatively excited a person is
Concentration	Indicates how concentrated the person is
Arousal	Indicates deep and profound interest in the conversation
Extreme emotion	Indicates overall emotional activity
Cognitive activity	Overall cognitive activity
EmoCog ratio	Indicates rationality

The area under the ROC curve score for Nemesysco rages from 0.53 to 0.71 (Lacerda, 2009). However, this study did not clarify which version of Nemesysco was measured. However, some signals captured by QA5 have been validated such as ‘embarassment’ (Han and Nunes, 2010), stressed and arousal (Konopka et al., 2010 as cited in Mayew and Venkatachalam, 2010). Research has been conducted that have used the QA5 in the development of a conversational robot (Usui et al., 2008; Hashimoto et al., 2009). The guide to using QA5 states that noise and environment may influence results. In this study, this was controlled by ensuring a quiet background during interviewing.

To validate the signals used in this study, an open source software was used to correlate the vocal signals captured by QA5 with prosodic features extracted from Praat. Praat with is a voice extraction software which can be used to analyse, synthesize and manipulate speech (Boersma and Van Heuven, 2001). A correlation analysis was conducted to validate the features collected by Nemesysco Ltd. Vocal features extracted from Praat were pitch (mean and maximum), intensity (mean, energy, minimum, and maximum). Pitch is defined as the rate of the opening and closing of vocal folds, it is also known as fundamental frequency (Giles et al., 1979). Fundamental frequency and intensity are known to be important variables in communicating emotions in speech (Ramdinmawii et al., 2017). The average pitch value for male speakers are typically found to be 100–180 Hz and for females it is found to be 160–300 Hz. A high mean pitch has been associated with stress and arousal (Sondhi et al., 2015). Intensity is associated with the loudness of the voice and is associated with a variety of emotions including psychological stress (Van Lierde et al., 2009).

Table 2 shows that ‘stressed,’ ‘upset,’ ‘intensive thinking,’ ‘imagination,’ ‘energy,’ ‘excited,’ ‘emo cog ratio,’ ‘concentration,’ and ‘extreme emotion’ is consistent with prosodic features extracted in Praat which are consistent with the literature as described. Table 2 shows the correlation results.

**TABLE 2:** Correlation results between Nemesysco Ltd and a commonly used open source software.

Correlation results		

Feature	Sub-feature	LVA correlation
Intensity	Mean	Stress (*r* = 0.506, *p* = 0.002) Upset (*r* = 0.602, *p* ≤ 0.001)
	Energy	Stressed (r = 0.502, p = 0.002) Upset (r = 0.520, p = 0.002)
	Minimum	Stressed (r = 0.411, p = 0.016) Intensive Thinking (r = 0.352, p = 0.041) Imagination (r = 0.501, p = 0.003) Energy (r = –0.348, p = 0.044) Excited (r = –0.514, p = 0.002) EmoCogRatio (r = –0.388, p = 0.023)
	Maximum	Stressed (r = 0.435, p = 0.010) Upset (r = 0.499, p = 0.003) Imagination (r = 0.379, p = 0.028)
Fundamental frequency	Mean	Stressed (r = 0.534,p = 0.001) Energy (r = 0.742, p ≤ 0.001) Arousal (r = 0.471, p = 0.005) Concentration (r = 0.519, p = 0.002) EmoCogRatio (r = 0.641, p ≤ 0.001) Intensive thinking (r = –0.622, p ≤ 0.001) Imagination (r = –0.591, p ≤ 0.001)
	Maximum	Intensive thinking (r = –0.369, p = 0.032)

To record voice analysis during interviews a Zoom H4N Pro Handy Recorder was used to record the voice signals. The voice of the interviewer was edited out using Audacity software version 2.1.1 prior to post-processing of the participant’s voice using Nemesysco Ltd’s QA5.

#### Honest Signal Detection

Pentland and Heibeck (2010) propose that there are four honest signals which are present in all social interactions and reveal a persons unconscious attitudes; (1) mimicry, (2) consistency, (3) activity, and (4) influence. Sociometric badges were developed by Pentland to detect a range of signals hypothesised by Pentland to relate to ‘honest signals.’ (see Pentland and Heibeck, 2010 for more in depth discussion). Sociometric badges have been used to detect signals in dyadic interactions (Paxton et al., 2015; Zhang et al., 2018b; Holding et al., 2019). The ROC score for these badges have been reported at 0.8 (Zhang et al., 2018b).

Honest signals are detected by four sensors: a microphone, an infrared sensor, a Bluetooth detector and a motion detector (Olgui̇n and Pentland, 2007). The microphone detects vocal tones and not content (Table 3, Features L – U). The infrared sensor captures movement relative to other interlocutors (Table 3, Features E, F, J, K). The Bluetooth sensor detects other badge wearers. Each badge is around the size of an identity badge and is worn around the neck. Table 3 lists the signals which can be extracted from the sociometric badge data.

**TABLE 3:** Definitions of signals produced by Sociometric Badges.

Feature	Description
A) Body movement	Normalised acceleration magnitude over 3 movement axis
B) Body movement activity	Absolute value of the first derivative of the accelerometers energy
C) Body movement rate	Indicates the direction of change in activity level (compared to first derivative)
D) Body movement consistency	Movement consistency throughout interaction
E) Body movement mirroring	Mimicking of other badge wearers body movement
F) Body movement mirror lag	Delay in mimicking of body movement
G) Posture front back	Orientation of front back panel
H) Posture activity	Absolute angular velocity
I) Posture rate	Angular acceleration
J) Posture mirroring	Mimicking of other badge wearers posture
K) Posture mirror lag	Delay in mimicking of posture
L) Successful interruptions	Number of successful interruptions made by the badges wearer
M) Unsuccessful interruptions	Number of unsuccessful interruptions made by the badge wearer
N) Speed of turn-taking	Indicates speed of turn-taking in a conversation
O) Overlap	Total amount of speaking whilst someone else is also speaking
P) Total speaking	Total amount of combined speaking (speaking and overlap combined)
Q) Volume front	Average absolute value of amplitude of the front microphone
R) Volume consistency front	Measurement of change in speech volume
S) Front pitch	Pitch of the voice from the front mic correlated with the fundamental frequency of the voice signal
T) Volume mirroring	Mimicking of other badge wearers volume
U) Volume mirroring lag	Delay in mimicking of other badge wearers volume

Sociometric badges were worn by both the participants and interviewers during interviews. After the interview the data stored locally on the badges were exported as structured meetings (as participants were facing each other in a single meeting) with a resolution of 1 s intervals (Sociometric Solutions, 2015). Badges worn by the trainer and the participant were synced using Sociometric Solutions software (Sociometric DataLab Enterprise Edition 3.1.2824).

### Facial Expression Detection

Facial expressions were detected using iMotions Biometric Research Platform 6.4 software and analysed using Affdex by Affectiva. This commercial software uses an emotional facial Analysis Coding System (EmFACS) that produces 7 facial expressions (sad, joy, anger, fear, disgust, contempt, and surprise) that humans use to communicate (Ekman and Friesen, 1971). Brow furrow, smirking and smiling were also assessed as they are considered important for a media interview (Taylor, 2015). Affdex by Affectiva’s ROC score has been reported as 0.8 for joy, disgust, contempt and surprise (Dupré et al., 2018). Interviews were recorded using a Sony PJ220 handycam camera. Any edits on the recordings were done using Adobe Photoshop. The video recordings were then imported into iMotions and post-processed using Affdex.

### Hand Movements/Gestures Detection

The Shimmer 3 Unit+ was used to capture hand movements. The Shimmer device contains a 3-point (x,y,z) direction accelerometer which was used to obtain an estimate of hand movements used during interviews which use of hand gestures will be inferred.

### Sequence of Events and Timestamps

All recordings of communication channels were synchronised to 1 s timestamp due to the capabilities of the different technologies. Some technologies were not able to record shorter timestamps. The data were analysed as if displays of social signals occurred simultaneously within the 30 s time frame (Paxton et al., 2015; Naim et al., 2016; Zhang et al., 2018a; Holding et al., 2019; Pereira and Hone, 2021).

### Subjective Measures of Communication Skills

Evaluation of participants communication performance by human raters was important as this would reduce bias when identifying effective and poor communicators. An evaluation was also done to identify relationships between patterns of emotional/non-verbal signals and trainee performance evaluations, as rated by humans. To obtain objective judgements of trainees’ performance, participants interviews were rated by the trainer and later, three neutral observers using a communication evaluation questionnaire (see section “Conversation Skill Rating Scale”).

There were several approaches taken to reduce the subjectivity in the ratings of performance. Firstly, because trainers had interacted with the trainees on the day of training which would have likely influenced their scores as a result of an interaction impression that could influence judgement ratings (Meissel et al., 2017), additional ratings were obtained by three neutral observers who were not present on the day of training (Naim et al., 2016). Ratings obtained from three neutral observers were intended to act as an audience by being able to review both interviews multiple times for a more thorough rating as well as provide more realistic ratings (Naim et al., 2016). Secondly, to further reduce the potential to rating bias the neutral observers were blind to the ratings provided by the trainer.

#### Trainers and Neutral Observers

The journalists in the first workshop were male and female who had more than 20 years field experience and had conducted the first media skills training workshop. The journalists in this workshop had provided feedback to participants about their performance following their interviews. Interviews were split equally between the two journalists. Both the journalist and the neural observers were able to playback and pause their interviews. The journalist that conducted interviews in the second workshop was a female with 4 years field experience and had conducted all interviews.

The neutral observers recruited to rate communication performance from camera recordings were not trained on what is considered ‘effective communication’ and were treated as a member of the general population. The three neutral observers recruited for the first workshop were different to the neutral observers for the second workshop. Neither the journalists or the neutral observers knew who had been labelled as an effective communicator or a poor communicator.

#### Conversation Skill Rating Scale

Subjective human ratings of communication skill was obtained using the Conversation Skill Rating Scale (CSRS) (Spitzberg and Adams, 2007). The CSRS has two rating sections; a 25-item scale rating verbal and non-verbal communication features and a 5-item scale measuring overall communication performance (molar ratings). As this study included both a radio and a face-face interview, the overall communication performance scores (molar ratings) were used as this does not include any items from the scale that include interpersonal measures of communication which would not be visible to neutral observers when listening to the radio interview and thus cannot be rated. The raters were asked to focus on non-verbal cues while watching the videos.

The CSRS is a measure of interpersonal skills and is claimed to be applicable in ‘virtually all face-face conversational interactions’ (Spitzberg and Adams, 2007). Evidence for its reliability and validity has beene found in a number of settings including educational settings, job interviews and getting to know you conversations (Spitzberg and Adams, 2007). Although we have not found specific examples of its use in media skills assessment, neither were we able to identify any other validated tools claimed ot be relevant to this context. The internal reliability for the CSRS has consistently been above 0.85 and is often above 0.90. Inter-rater reliability has been assessed have found acceptable reliabilities above 0.75 (Spitzberg and Adams, 2007). The molar ratings were filled in by the trainers and three neutral observers to rate communication skills performance in the on-camera interviews.

### Procedure and Media Skills Workshop Details

The study took place on the campus of a London University within the context of two a media training days for researchers. The first three workshops were conducted by media training professionals with over 20 years of professional experience of journalism. A total of three training days took place within the period April 2017 to June 2017 with the number of participants attending each day ranging from 5 to 6. All data collection took place in a standard university seminar room with tables, chairs, and a projector. The remaining three workshops were conducted by an early career journalist with 6 years experience in the field. These workshops took place with the period of November 2017 – December 2017. From this point forward, the journalists will be referred to as the trainers.

Prior to attending the training, participants were asked to provide a brief summary of their research that is comprehensible to a non-specialist population, including importance and worst anticipated question in a media interview. This was to help the trainers prepare for conducting practice media interviews tailored to the individual participants’ research profiles and work.

On arrival at the training day, participants were fully briefed on the study and formal consent was collected, along with demographic information (job role, gender, age and ethnicity, presence of social/communication disability, and prior experience of presentation). If participants did not wish to give consent to the recording of social signals, they were given the option to participate with the systems switched off during interviews without penalty. All participants gave consent to record signals.

**FIGURE 1:**
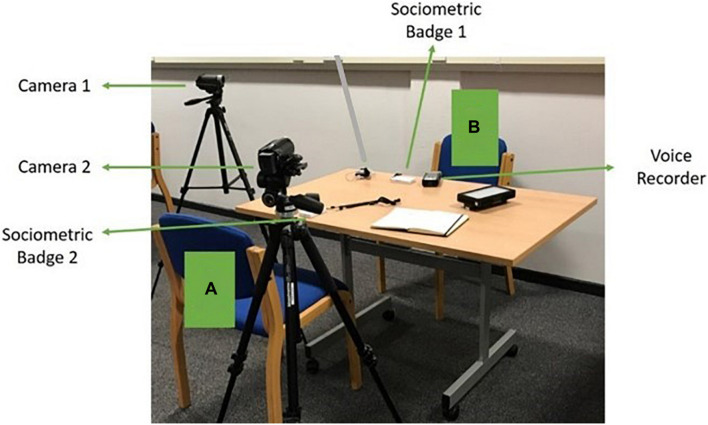
Study layout of both radio and on-camera training sessions. Cameras were only turned on for recording during the on-camera interview. **(A)** Journalist position. Both cameras are facing the participant **(B)** for more accurate post hoc face recognition. The added voice recorder was for a better-quality recording of interviews.

After an introduction, participants took part in a 45 min to 1-h lecture style introduction to effective media interview communication skills. The lecture was presented in a group setting. Participants were then given individual time slots during the day to come back to do practice interviews with the trainers. The practice interviews were conducted individually, and two practice interviews were conducted for each participant. The first was to simulate a radio interview, so the participants sat face-to-face with a voice recorder on the table. No cameras were turned on during interviews to avoid any influence this may have on performance. The second practice interview was a simulation of an on-camera interview, so the camera was located behind the journalist and beside the participant. Participants were informed that the camera was placed behind the journalist was recording as if for a broadcast. The room set up is illustrated in Figure 1. One of the trainers acted as the interviewer for the purpose of the practice media interviews.

Prior to commencement of the interviews, participants were connected to a Shimmer 3 GSR device and both the participant and interviewer put on a sociometric badge. The room was also set up with further recording equipment to allow social signals/emotion detection as shown in Figure 1.

During the practice interviews, participants were asked individually relevant questions about their research. The first question asked participants to explain their research. These questions were based on the material supplied by participants. The question difficulty was pitched to increase as the interviews progressed. Each interview lasted between 5 and 8 min.

Interview recordings were played back to participants after each interview and they were then provided performance feedback by trainers who were able to playback interviews which enabled the trainer and trainee to pause and rewind the video for effective performance feedback. Trainers were then asked to fill in the CSRS which is a standardised measure of communication skill.

Following completion of the study participants received a short closing statement reminding them of the purpose of the research. Participants were reimbursed £5/h for recognition of their time.

The subjective nature of human judgement makes the ground truth for interviews hard to establish. The trainer interacted with the trainers during the lecture, during the interview and provided feedback after each session. This amount of interaction may have had an influence on the trainers’ ratings. Therefore, in order to remove potential bias, the recorded interviews were subsequently rated by three neutral observers also using the CSRS. Further benefits of this is that the neutral observers could review the material multiple times which enables them to rate the interviews more thoroughly. The video recordings from on-camera interviews were presented to neutral observers to obtain judgements of communication performance. Ratings from these observers were likely to be similar to audience ratings of a media interview, as opposed to expert ratings (Naim et al., 2016). Neutral observers were able to interact with the videos by pausing, rewinding and forwarding the videos of each participant. Each neutral observer worked individually and was blind to the ratings provided by others.

This research was conducted in accordance with the Declaration of Helsinki and ethical approval was obtained from the Ministry of Defence Research Ethics Committee as well as the Universities Research Ethics Committee.

## Results

### Subjective Ratings of Communication Skills

#### On-Camera Interview

Participants’ communication was rated using the CSRS by the trainers and later, three neutral observers. Internal consistency was calculated using Cronbach’s Alpha. Then, a composite mean of the overall communication skills rating (based on the five molar ratings) was obtained for trainer ratings and three neutral observers. Inter-rater reliability was conducted to calculate agreement between the raters using intraclass correlation with a two-way mixed approach (Mandrekar, 2011). The internal consistency was high for communication ratings for all raters of communication (molar scores, n = 5) was α = 0.95. The intraclass correlation was 0.78 with a 95% confidence interval from 0.603 to 0.870 [F_(4.289)_, p < 0.001]. This moderate agreement warrants a weighted average (Mandrekar, 2011). A median of the dataset was 24.33 which established effective (M = 28.35; SD = 3.22) and poor communicators (M = 19.60; SD = 3.10).

### Social Signal Displays During Communication

#### Missing Data

Cases with missing data from any channel were excluded from the analysis. A total of six participants were excluded (three due to low quality video recordings for facial expression, two due to missing hand gesture data, and one due to missing sociometric badge data). This resulting in a sample size of 33 participants included in the analysis.

#### Data Preprocessing – Normalisation

The social signal data was normalized using the minimum and maximum values of the datasets resulting in a dataset range from 0 to 1 (Gao et al., 2012). The formula can be seen below.


$$x^{\prime }=\frac{x-\min\left(x\right)}{\max\left(x\right)-\min\left(x\right)}$$

#### Thin Slices of Behaviour

Research has found that the first 30 s of an interaction was most effective in assessing judgements and perceptions about people as raters of performance base their scores on the initial stages of an interaction (Sullivan, 2018). Impressions are typically made during this time scale even though a full interaction may take place. This suggests that the interviewees’ response to the first question in the interview could have swayed observers in forming initial judgements about their communication abilities. It is for these reasons that we decided to investigate the first 30 s of the recorded interviews. In addition to this, in an interview context it has been found that the first 30 s are pivotal for making a decision about candidate as rapport is built in the first 30 s (Forbes and Jackson, 1980; Duggan and Parrott, 2001).

The first 30 s of a media interview are beneficial for establishing patterns in social signals that are associated with media interview performance judgement. The first 30 s in the interviews were enough to obtain the first question and response in each interview. As noted previously, research has shown that the first part of interview allows interviewers to make a judgement/form an impression of the interviewee (Sullivan, 2018). The same can be said for media interviews (Taylor, 2015), public speaking (Chollet et al., 2015), how our speaking behaviour predicts how we are perceived in online social multimedia (Park et al., 2016) and in job interviews (Nguyen and Gatica-Perez, 2015; Naim et al., 2016). Additionally, a meta-analysis has found that prediction ratings do not differ between 30 s of the interview and 5 min (Ambady and Rosenthal, 1992).

#### Machine Learning Classification Techniques

##### Establishing a ground truth

An average of the neutral observers (judges) ratings were obtained for each participant (Naim et al., 2016). The neutral observers ratings were collected as they were treated as an audience.

A median for the dataset was identified so that each interview could be labelled as effective or poor communicators forming a ground truth for machine learning techniques that will be used. This was done to establish a mid-point in the dataset to establish high and low ratings of communication. A high value indicates effective communication and a low value indicates poor communication. The cut-off between good and bad in the on-camera interview was 24.33. Radio ratings were not included.

##### Feature selection

**FIGURE 2:**
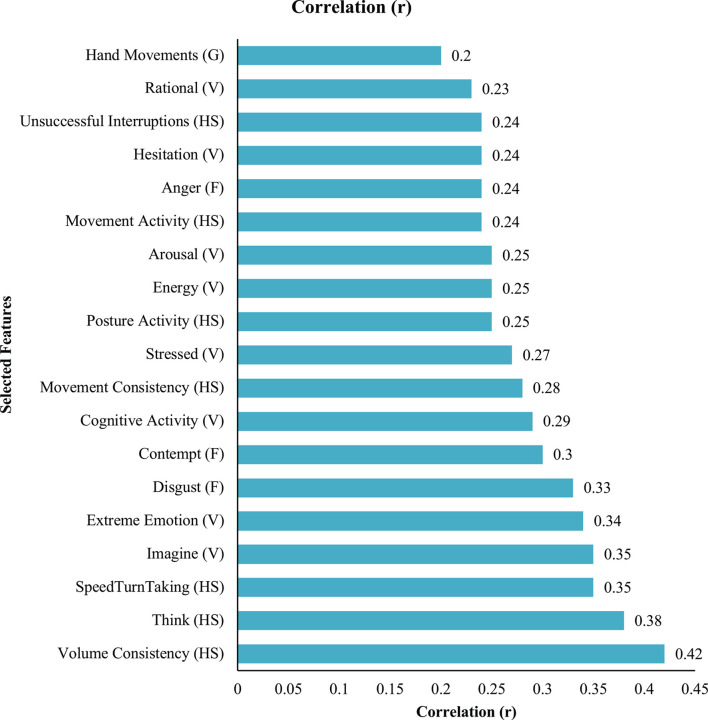
Features selected for inclusion in machine learning classification. HS, honest signals; F, facial expression; V, vocal behaviour; G, gestures.

The relationships between patterns of non-verbal signals and trainee performance evaluations were explored using Weka GUI Version 3.8. Features were selected using a correlation-based features selection (CFS) anything below a cut-off point of 0.2 was excluded. This method selects the features which are highly correlated with the labelled data and uncorrelated with each other (Witten and Frank, 2002). CSF was applied to all the communication channels simultaneously. The CSF method was used for features selected for inclusion in machine learning analysis in which a binary classification of good and bad communication ratings. The features selected based on the CSF methods can be seen in Figure 2.

#### Machine Learning Classification

Using the collected and preprocessed data, performance was evaluated using the following classifiers (used with default parameters unless stated otherwise) Logistic Regression, Naïve Bayes, Decision Tree, k-Nearest Neighbour with a parameter where *k* = 3 and Support Vector Machine (Poly Kernel). The number of participants who were classified as effective communicators were (15) and the number of participants that were classified as poor communicators were (18).

Leave one out cross validation has been used for unbalanced data as well as data with a small sample size (Witten and Frank, 2002). Leave-one-out cross-validation is where the algorithms applied once for each instance, using all other instances as a training set and using the selected as a single-item test set (Witten and Frank, 2002). The *F* measure (also known as F1 score or *F* score) was selected as the performance evaluation metric as it is well suited for imbalanced classification data and it combines both precision and recall (Goutte and Gaussier, 2005). Analysis was done using Weka 3.8.4. According to Table 4, the best result for the current dataset is the naïve Bayes which produced an accuracy score of 78%, a F-measure of 0.76.

**TABLE 4:** Machine learning classification results.

Machine learning classification algorithm	Accuracy	*F*-Measure (weighted average)	ROC (weighted average)
Logistic regression	61%	0.60	0.59
**Naïve Bayes**	**78%**	**0.76**	**0.79**
Decision tree	55%	0.51	0.50
k-Nearest Neighbours (*k* = 3)	67%	0.64	0.66
Support vector machine (PolyKernel)	64%	0.63	0.62

*Bold highlights the best result.*

### Social Signal Display – Differences Between Groups

**FIGURE 3:**
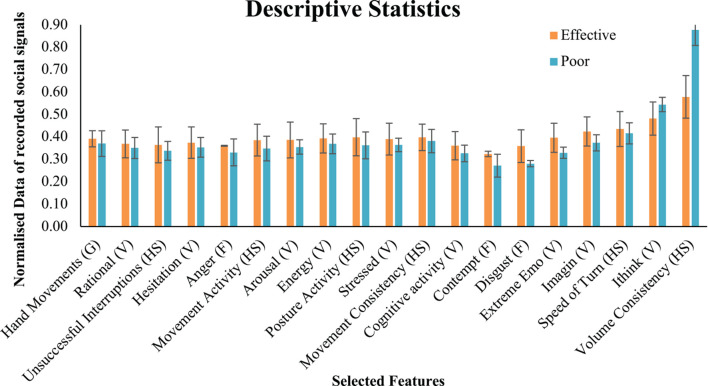
Descriptive statistics for social signals. HS, honest signals; F, facial expression; V, vocal behaviour; G, gestures. The normalised data is the data from social signals captured by the automated technology.

Descriptive statistics for effective and poor communicators for each social signal can be seen in Figure 3. The error bars that are displayed on the table are standard error.

A more formal statistical analysis was conducted to test if the individual selected signals differed between effective and poor interview ratings performance. A Mann–Whitney *U* test was used to assess differences between group displays of features. Results found that *anger* and *movement consistency* was significantly different displayed between effective and poor communicators. Where those who communicated more effectively displayed more *anger* than those who performed poorly according to neutral observers. Those who were rated as more effective communicators displayed more consistent movements than those who were poor communicators. The results can be seen in Table 5.

**TABLE 5:** Man–Whitney *U* test results.

Signal	Mann–Whitney *U*	Sig (*p*-value)
Hand movement (G)	110	0.366
Cognitive activity	100	0.206
Unsuccessful interruptions	100	0.196
Hesitation	101	0.219
Anger	71	0.021*
Movement activity	95	0.148
Arousal	118	0.539
Energy	89	0.100
Posture activity	99	0.193
Stressed	104	0.262
Movement consistency	59	0.006*
Rationality	111	0.386
Contempt	96	0.159
Disgust	115	0.470
Extreme emotions	90	0.104
Imagination	91	0.116
Speed of TurnTaking	87	0.084
IThink	90	0.104
Volume consistency	89	0.096

*Less than 0.05.

## Discussion

Research in the field of video observation to understand social interaction is subjective and does not scale with large data. Automated technology may present as a possible solution by objectively detecting non-verbal signals and doing so much faster than manual coding of observational data. As a result of this problem, the aim of our research was to explore which combinations of social signals are most promising for automatically evaluating trainee performance. The results suggest that body positioning, facial expressions, vocal signals and hand gestures are all relevant for the context of media interviews. Combinations of these signals were produced a prediction of good and bad performances with an accuracy of 78% and an F-measure of 0.76. Two social signals suggested that there was a difference between effective and poor communicators. Effective communicators displayed more anger and more consistent movements in the first 30 s of their interview than those who were identified as poor communicators. The findings of the study are presented in more detail in the sections which follow and discussed in light of previous research.

### Honest Signals

Honest signals included in the feature selection for the formal multimodal analysis included unsuccessful interruptions, movement activity, posture activity, movement consistency, speed of turn taking, and volume consistency. Formal analysis of the results revealed that movement consistency was significantly different between groups where those who were rated as effective communicators.

Previous literature has found that consistency in movement suggests that the communicator is relaxed, calm and confident. This is particularly important in media interviews as too much fidgeting can suggest the interviewee is uncomfortable (Taylor, 2015). This is a level of consistency which could have been identified by the judgers as an effective method of communicating (Hill et al., 1981).

### Vocal Behaviour

The vocal signal, as labelled by Nemesysco, included in the analysis was cognitive activity. Descriptive statistics suggested that effective communicators displayed more cognitive activity. However, this difference was not significantly different. This result does suggest that overall thoughtfulness in vocal behaviour is important in the context of media interviews in the first 30 s. During the first 30 s of the interview captured the first question asked by the journalist which suggests that the interviewers were thoughtful in their response to this initial question. Reasons why this may not be significantly different could be a result of the fusion analysis, i.e., vocal displays of thinking in combination of another social signal.

### Facial Expressions

The facial expressions identified in this study as a predictor of effective or poor communication in the context of media interviews are *anger* and *disgust*. Results suggest that those that were classified as effective communicators by neutral observers displayed more anger and more disgust than those who were classified as poor communicators.

The AU involved in *anger* facial expression are AU4, AU5, AU7, and AU23. The AUs involved in *disgust* are AU9, AU15, and AU16. AU9 and AU4 are both associated with the lowering of the brow. This lowering of the brow has been associated with concentration. As the data were only analysed for the first 30 s of the interview, this could suggest that participants were listening to the first question posed by the journalist or they were concentrating (Ekman, 1997).

### Hand Movements/Gestures

Hand gestures were included in the feature inclusion analysis. This suggests what the literature has informed us, that gestures assist in communication (Goldin-Meadow and Alibali, 2013). These results suggest that wearables could be used to support media presenters as it is a low cost intervention for capturing hand gesture use. Interestingly, Damian et al. (2015) developed a system for providing real time feedback during public speaking based partly on gesture capture. This design choice was driven by the practical consideration of what would work in a potential noisy environment and did not include pre-testing for what predicts ‘good’ performance. However, our findings provide some empirical support for their chosen approach.

### Combinations of Signals

The combination of social signals included hand movements (G), rationality (V), unsuccessful interruptions (HS), hesitation (V), anger (F), movement activity (HS), arousal (V), energy (V), posture activity (HS), stressed (V), movement consistency (HS), cognitive activity (V), contempt (F), disgust (F), extreme emotion (V), imagination (V), speed of turn taking (HS), thinking (V), and volume consistency (HS). The signals included in the analysis as a result of the feature inclusion analysis included honest signals, facial expressions, hand gestures, and vocal behaviour. This suggests that communication during media interviews are multimodal which has been suggested numerous time (Pantic et al., 2011; Bekele et al., 2013; Potamianos, 2014; D’mello and Kory, 2015; Esposito et al., 2015; Hunyadi, 2019).

Detection of contempt and anger facial expression could suggest a false positive as people often frown when listening to someone. Additionally, brow furrow is an AU that makes up contempt. It does not mean that they are angry but it could be a sign of concentration (Rozin and Cohen, 2003). This is consistent with the inclusion of the ‘cognitive activity’ feature in the feature selection process suggesting that participants were listening (frowning and contempt) and responding in a thoughtful manner.

### Current Study Limitations and Future Work Recommendations

This exploratory study had a relatively small sample size of 33 participants. An increase in sample size in future work would be helpful to test the reliability of the findings described here. In addition to this, a larger sample size would enable the investigation of gender differences and to assess whether there are any cultural differences in performance. Results should therefore be interpreted with caution.

The study looked only at one population composed of early career researchers within a university setting. While to some extent this population can be seen as representative of the kind of professional role where employees may be called upon to engage in media interviews, it would be interesting to confirm the findings for trainees in other organisation types. None of the trainees were expert at media skills which could have restricted the range of performance. It would be interesting in future work to include expert as well as novice participants. However, the findings are relevant for a training context where trainees are usually not already experts. Participants also received different questions from one another given their own research background. While this increased the ecological validity of the study, it reduced the degree of experimenter control over stimuli and may have led to differences in difficulty and/or emotional impact across different participants. However, we included the first 30 s of the interview in the analysis which would have included the initial question which was for participants to describe their research. Future work could potentially look to explore the use of more standardised question sets.

A limitation related to hand gesture detection was that the technology of gestures was strapped to the non-dominant hand which may not be a true representation of hand gestures. However, the results found in this study are consistent with previous research suggesting that use of hand gestures are often perceived by others as effective communicators. Future research could explore the use of non-contact detection of gestures to avoid this issue.

A potential limitation for this research is that the entire video was shown to neutral observers while the first 30 s were included in the analysis. However, research has shown that there were no differences in predictions based on 30 s or 5 min (Ambady and Rosenthal, 1992). However, future research could explore and verify this.

A final limitation of this paper is that the verbal content could have influenced the “effective communication” ratings that neutral observers gave participants. However, since the researchers have included vocal behaviour as a variable the content could not have been made unintelligible (i.e., random splicing or filtering) as this would not have been suitable for the aims of this paper. Moreover, the raters were instructed to focus on non-verbal features when rating communication effectiveness.

There is emerging evidence of the added value of combining signals across multiple modalities to improved classification accuracy (e.g., Pantic et al., 2005; Turk, 2014). The approach described in this paper facilitates understanding of the value and the insights that can be gained from each tool and as such is most relevant to providing feedback to interviewees, since they would need to know which signals from each tool to focus on to improve performance. Future work could compare results from different tools and assess appropriateness of each to the current setting.

The use of off-the-shelf-commercial-technology has limitations in that the algorithms used to classify non-verbal behaviour are not open to direct scrutiny by researchers. Nevertheless, the results found were consistent with previous literature which supports their practical applicability in this domain which validates the current results.

Another possible limitation for this research is that the first 30 s were evaluated only. Details surrounding trainees communication performance could have improved or worsened over the course of the interview which was not included in this analysis. However, research has shown that judgements or impressions of performance are decided in the first stages of the observation and performance throughout the remainder of the interview are treated as confirmation of initial judgements made (DeCoster and Claypool, 2004; Sullivan, 2018). Additionally, the first 30 s were also used to control the initial interview questions to control constraints around questions and to remain consistent for all the participants. Future research could investigate the whole interaction instead of the first 30 s.

## Conclusion and Contributions

In this paper we investigated whether social signals can be detected in a dyadic interaction using commercial automated technology and whether good interviews could be distinguished from poorer interviews on the basis of such signals. The findings from this research illustrate that several commercial technologies are capable of detecting performance-relevant social signals in a media interview where there is a reciprocal exchange of social signals.

The results from this research have potential application in a range of contexts. They could be used to assist trainers in conventional media skills training by giving them a mechanism for providing trainees with more objective feedback about their non-verbal performance to enhance their communication skills. Our results can help trainers choose the most useful off-the-shelf-technologies to use to support their role and highlight the most relevant signals to provide feedback on. The results suggests that for on-screen interviews, honest signals are most prevalent signal necessary for media interview content, followed by facial expression. The technology used to capture hand gestures provides a good low cost alternative. The results could also be used to develop automatic training feedback systems to help learners self-reflect upon their performance. The results could also have relevance to researchers in fields such as journalism or social psychology conducting research requiring the assessment of media interview quality, since this could potentially be done automatically at a lower cost than using human coders. Finally, the results have the potential to inform the design of automated systems which could be developed to help in personnel selection or in employee appraisal for roles that involve a need to engage in regular media interviews.

## Data Availability Statement

The datasets presented in this study can be found in online repositories. The names of the repository and accession number can be found below: https://doi.org/10.6084/m9.figshare.11663487.

## Ethics Statement

The studies involving human participants were reviewed and approved by the Brunel Research Ethics Office and the Ministry of Defence Research Ethics Committee. The participants provided their written informed consent to participate in this study.

## Author Contributions

MP contributed to this research by conducting the research including data collection, recruitment, data-analysis, interpretation, and initial write-up of this manuscript. HM was provided the substantial advice on the different methods of analysing social signal data. KH designed the research who had also provided substantial contributions to the write-up of this manuscript, interpreting results, and provided advice on data-analysis. All authors contributed to the article and approved the submitted version.

## Conflict of Interest

The authors declare that the research was conducted in the absence of any commercial or financial relationships that could be construed as a potential conflict of interest.

## Publisher’s Note

All claims expressed in this article are solely those of the authors and do not necessarily represent those of their affiliated organizations, or those of the publisher, the editors and the reviewers. Any product that may be evaluated in this article, or claim that may be made by its manufacturer, is not guaranteed or endorsed by the publisher.

## Footnotes

1
Nemesysco.com
